# Ciprofloxacin and Metronidazole As Rare Culprits of Drug-Induced Immune Thrombocytopenia in the Setting of Diverticulitis Treatment

**DOI:** 10.7759/cureus.87835

**Published:** 2025-07-13

**Authors:** Jody W Tai, Ericka H Young, Vivek D Shah, Jarred P Reed

**Affiliations:** 1 Internal Medicine, Olive View University of California Los Angeles Medical Center, Los Angeles, USA; 2 Hematology and Medical Oncology, Olive View University of California Los Angeles Medical Center, Los Angeles, USA; 3 Oncology, Olive View University of California Los Angeles Medical Center, Los Angeles, USA

**Keywords:** ciprofloxacin, diverticulitis, drug-induced thrombocytopenia, immune thrombocytopenia, metronidazole

## Abstract

Drug-induced immune thrombocytopenia (DITP) is a syndrome in which antibodies bind to platelets only in the presence of a drug, leading to platelet destruction. A variety of medications, herbs, vaccines, and foods have been commonly associated with DITP. Here, we present a case of DITP in a 61-year-old female who was receiving two widely prescribed antibiotics, ciprofloxacin and metronidazole, which are not commonly linked to DITP. The patient developed thrombocytopenia within 24 hours of initiating these antibiotics, and her platelet count rapidly normalized following discontinuation of the drugs. Severe thrombocytopenia can lead to life-threatening bleeding; therefore, it is imperative that treating providers rule out alternative causes of thrombocytopenia and promptly discontinue any potential offending agents.

## Introduction

Immune thrombocytopenic purpura (ITP) is an acquired syndrome of thrombocytopenia caused by immune-mediated destruction of platelets, characterized by a platelet count of less than 100,000 per cubic millimeter (K/cumm). ITP may be classified as either primary or secondary. In primary ITP, sensitized immunoglobulin G (IgG) autoantibodies lead to T-cell-mediated destruction of platelets. Secondary ITP is commonly associated with underlying disorders that trigger platelet destruction, such as systemic lupus erythematosus, HIV infection, malignancy, or drug exposures, the latter being referred to as drug-induced immune thrombocytopenia (DITP) [1].

In DITP, naturally occurring antibodies bind with high avidity to platelet surface antigens, but only in the presence of a drug or its metabolite. Alternatively, the drug may bind to the surface of platelets, inducing a conformational change in antigens, which then stimulates antibody binding [2]. Diagnosing DITP is challenging, requiring the exclusion of alternative etiologies of thrombocytopenia while obtaining a detailed history of newly initiated medications. It is often difficult to identify the responsible drug in individuals with multiple concomitant medical conditions that may also contribute to thrombocytopenia.

We present a case of a patient with no prior history of thrombocytopenia who had visited urgent care a few days earlier with abdominal symptoms, including nausea. She was prescribed empiric treatment for uncomplicated acute diverticulitis. Within one day of starting the medication, she presented to our emergency department with profound thrombocytopenia. After ruling out alternative causes and based on the clinical course, we attributed the rapid decline in platelet count to recent antibiotic exposure, specifically ciprofloxacin and/or metronidazole, two agents that are uncommonly reported as causes of DITP.

## Case presentation

A 61-year-old female with a past medical history of hyperthyroidism and anemia presented to the emergency department in January 2025 with loose stools and diarrhea accompanied by left lower quadrant pain. Stool cultures were negative for bacterial or viral causes, including Clostridium difficile, and no abdominal imaging was obtained at that time. CRP was within normal limits. She was empirically prescribed ciprofloxacin 500 milligrams (mg) twice daily and metronidazole 500 mg three times daily for a 10-day course to treat a presumed diagnosis of diverticulitis. Laboratory testing at that time showed a normal platelet count of 318 K/cumm (reference range 160-360 K/cumm).

Approximately 12 hours after initiating the medications, she developed palpitations, chest pain, and dyspnea. At the recommendation of her local urgent care, she presented to our emergency department on the fourth day of taking both ciprofloxacin and metronidazole. Cardiac workup was unremarkable for acute coronary syndrome. Physical examination revealed only reproducible left lower quadrant abdominal pain without rebound tenderness or guarding. She had no signs of mucocutaneous bleeding, bruising, or rash. She denied melena, hematochezia, fever, night sweats, or unintentional weight loss.

Laboratory investigations, including a CBC and other relevant tests, were performed. These results are summarized in Table 1. 

**Table 1:** Laboratory results on admission. Hgb: Hemoglobin; MCV: Mean corpuscular volume; LD: Lactate dehydrogenase; PT: Prothrombin time; INR: International normalized ratio; PTT: Partial thromboplastin time.

Test	Result	Reference Range
WBC	5.7 K/cumm	4.5-10.0 K/cumm
Hgb	11.7 g/dL	12.0-14.6 g/dL
MCV	90.5 fL	82-97 fL
Platelets	8 K/cumm	160-360 K/cumm
LD	125 U/L	98-192 U/L
PT	14.4 sec	11.9-14.6 sec
PTT	26.9 sec	22.9-33.1 sec
INR	1.12	0.87-1.14
Folate	14.9 ng/mL	>7.0 ng/mL
B12	371 pg/mL	213-816 pg/mL
HIV	Nonreactive	Nonreactive
Hepatitis A IgM	Nonreactive	Nonreactive
Hepatitis B IgM	Nonreactive	Nonreactive
Hepatitis B surface antigen	Nonreactive	Nonreactive
Hepatitis C antibody	Nonreactive	Nonreactive

Workup for gastrointestinal infection with stool cultures was negative; studies for nutritional deficiencies were within normal range. Laboratory tests, including coagulation studies for thrombotic microangiopathy (TMA), were not suggestive of pathology. Autoimmune screening showed a low-positive antinuclear antibody (ANA) titer of 1:40.

**Figure 1:**
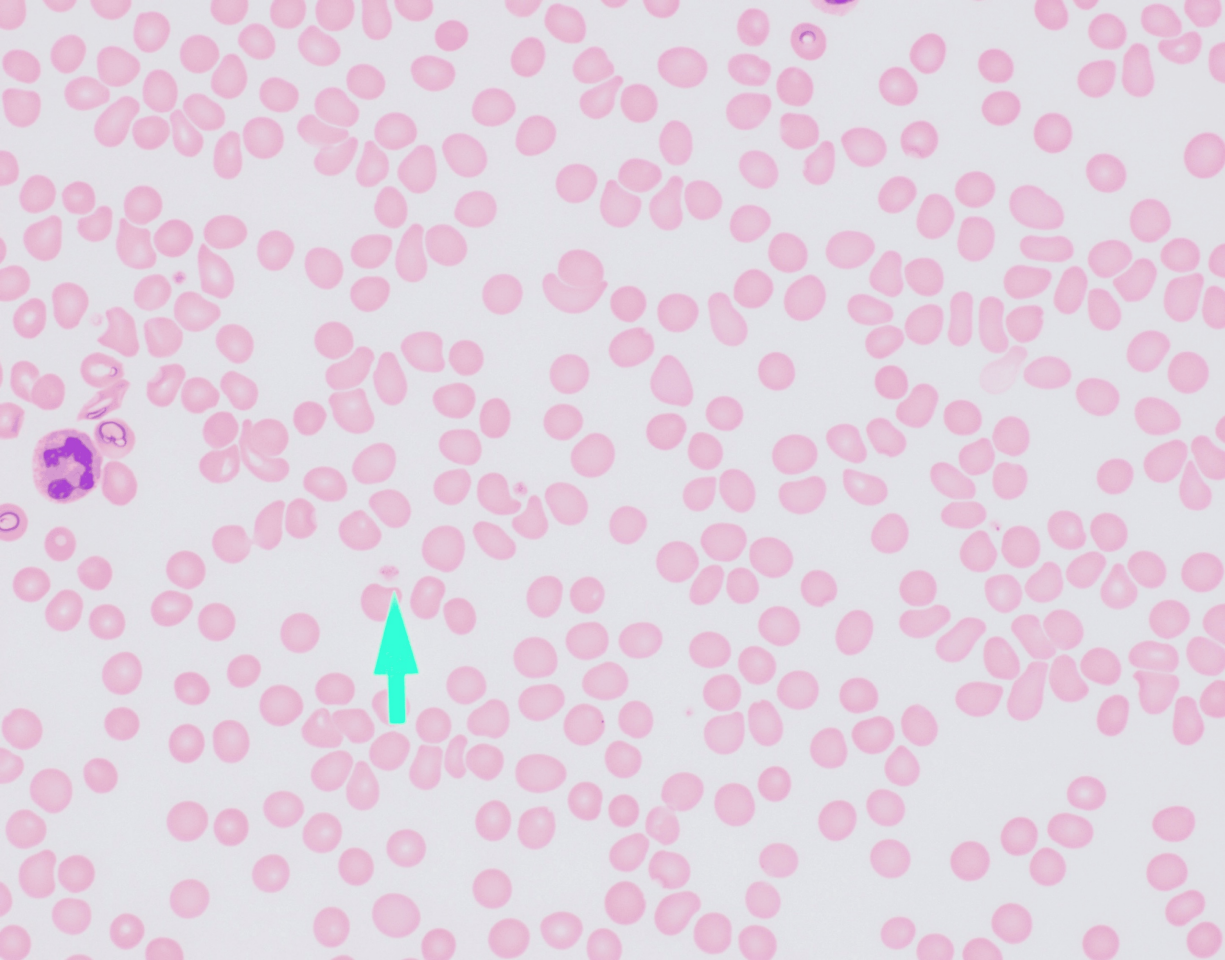
Peripheral blood smear visualized at 600× magnification, showing an overall reduced number of platelets. The green arrow indicates a platelet with normal morphology.

Peripheral smear review demonstrated markedly reduced platelets without clumping, consistent with laboratory findings (Figure 1).

**Figure 2:**
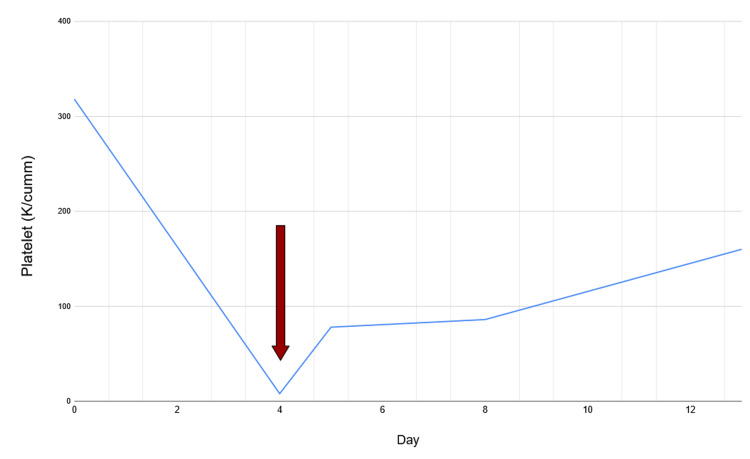
Platelet trend during and after hospitalization. Day 0: Platelet count at 318 K/cumm prior to initiation of metronidazole and ciprofloxacin. Day 4 (red arrow): Platelet count dropped to 8 K/cumm. Day 8: Platelet count increased to 86 K/cumm on the day of discharge. Day 13: Platelet count normalized to 160 K/cumm at post-hospitalization follow-up.

A CT scan of the abdomen and pelvis showed no acute intra-abdominal pathology. Based on the clinical presentation, the likely etiology was ITP, either infection-related or drug-induced. There was low suspicion for disseminated intravascular coagulation (DIC) or TMA given normal hemolysis markers. As part of the diagnostic algorithm, all recently initiated medications, including ciprofloxacin and metronidazole, were discontinued. Since gastrointestinal symptoms had nearly resolved by the time of presentation, no alternative antibiotics were prescribed. During the course of the workup and management, one unit of platelets was transfused, and the patient’s platelet count increased from 8 K/cumm to 78 K/cumm within two hours post-transfusion. The patient’s platelet trend during hospitalization is summarized below in Figure 2. Day 0 represents the day of initial presentation with loose stools and diarrhea, upon which she was prescribed and began taking ciprofloxacin and metronidazole. Day 4 marks her presentation to our emergency department, and Day 8 corresponds to the final day of hospitalization. Day 13 reflects the normalization of her platelet count to 160 K/cumm at the post-hospitalization laboratory follow-up. Of note, the patient did not receive steroids or intravenous immunoglobulin (IVIG) during this period.

At the time of writing, her platelet count had normalized to 275 K/cumm at the most recent follow-up visit with her primary care physician, four months post-discharge.

## Discussion

Only 20% of adults diagnosed with ITP have the secondary form, with a prevalence of approximately 12 per 100,000 individuals [3]. However, DITP is an even rarer cause of secondary ITP, with an estimated incidence of 10 cases per million in the US and Europe [4]. The most common offending drugs include heparin, antibiotics such as quinidine and trimethoprim-sulfamethoxazole, ibuprofen, and glycoprotein IIb/IIIa inhibitors. To our knowledge, there have been fewer than 10 reported cases of ciprofloxacin-induced DITP, involving both intravenous and oral formulations [5-6]. Most of these cases were in patients with urinary tract infections and resolved upon cessation of the medication. Notably, the use of other fluoroquinolones is not necessarily associated with cross-reactivity following ciprofloxacin-induced DITP [6-8].

We are aware of only one reported case of DITP attributed to metronidazole, also involving intravenous administration. That case demonstrated spontaneous recovery in platelet counts after cessation of the offending agent, confirmed with drug-dependent antibody testing [9]. Overall, drug-dependent platelet antibodies have been demonstrated for both metronidazole and ciprofloxacin, but clinical case reports remain scarce.

Diagnosis of DITP can be difficult because of the temporal relationship with drug initiation, variable presence or absence of bleeding, multiple comorbidities that hospitalized patients may present with, and preexisting polypharmacy, all of which can confound identification of the true drug culprit. The diagnosis of DITP should be suspected in cases of severe acute-onset thrombocytopenia, typically with platelet counts less than 20 K/cumm, occurring approximately five to ten days after the initiation of a new medication [10]. In cases where the diagnosis is challenging, such as in those with multiple concomitant potential etiologies of thrombocytopenia and polypharmacy, testing for drug-dependent antibodies by flow cytometry can theoretically be of utility, though this typically requires sending blood samples to a specialized laboratory [11]. These tests evaluate antibody binding to normal platelets in the presence of both the patient’s serum and potential culprit drugs. If negative, the diagnosis of DITP is not ruled out for several reasons: 1) antibody titers may decline depending on the timing of blood sampling; 2) drug metabolites rather than the parent drug may be responsible for the immunogenic response; or 3) the platelets used for testing may have surface antigens that differ from those present in vivo [11]. Though a positive result can guide management in determining which drug to discontinue, a negative result should not delay drug discontinuation. Clinical suspicion should take precedence in decision-making.

We utilized a platelet transfusion to aid in both therapeutic and diagnostic efforts. We hypothesized that transfusion would result in an appropriate and durable rise in platelet count in the setting of drug discontinuation. Conversely, alternate etiologies of ITP would result in either no rise or only a slight rise in platelet count followed by a subsequent decline [12]. We avoided the use of steroids and IVIG, as their diagnostic utility would be lower than that of a transfusion. Specifically, steroids and IVIG could be therapeutic for either DITP or ITP of alternate etiologies [13]. Confirmatory platelet-antibody testing was considered but not performed due to high clinical suspicion for DITP and the patient’s rapid improvement.

**Figure 3:**
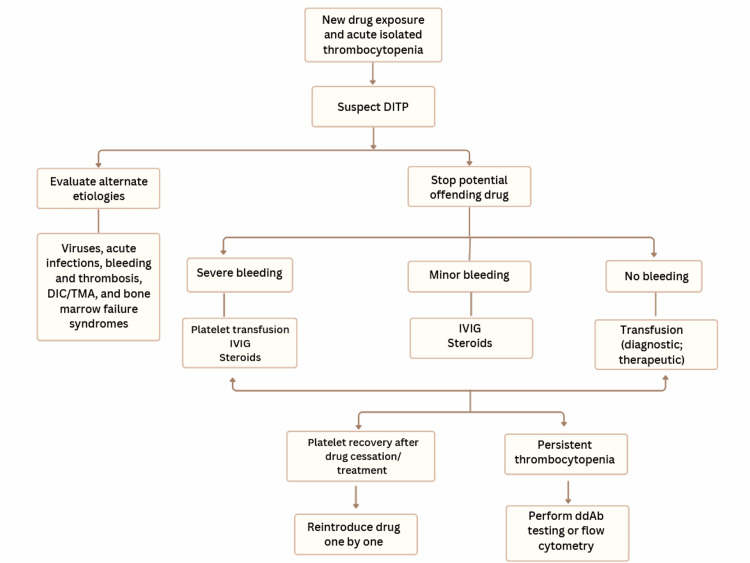
Algorithm for DITP evaluation and treatment. Once DITP is suspected in a patient presenting with a new drug exposure and acute isolated thrombocytopenia, alternative etiologies must first be excluded. If no alternative cause is identified, the suspected culprit agent should be discontinued immediately. Depending on the presence and/or severity of bleeding, clinicians can follow this algorithm for subsequent management steps, which may serve both diagnostic and therapeutic purposes. Figure created by the authors for the purposes of this article. DIC: Disseminated intravascular coagulopathy; TMA: Thrombotic microangiopathy; ddAb: drug-dependent antibody; IVIG: Intravenous immunoglobulin; DITP: Drug-induced immune thrombocytopenia.

A visual representation of the overall algorithm for DITP diagnosis and management is summarized in Figure 3.

In this patient’s specific case, there were no new exposures reported by the patient during the four days between presentations, apart from the two newly initiated medications. Since her thrombocytopenia began to improve after the discontinuation of the presumed offending agents without any other interventions, there was strong clinical suspicion that these medications were the causative agents, and thus a retrial of the medications was not attempted. In general, treatment for ITP may be supportive, with improvement in thrombocytopenia occurring within four to five half-lives of removing the offending agent [10, 14-15].

## Conclusions

ITP should be considered in patients who present with isolated thrombocytopenia, without evidence of hemolysis or bleeding suggestive of a consumptive coagulopathy. In such cases, secondary causes of ITP, including infection, malignancy, and medication use, must be ruled out. Although flow-cytometry testing for drug-dependent antibodies is the most definitive diagnostic method, in our case the marked rise in platelet count after discontinuing the suspected agents and administering a platelet transfusion provided indirect yet compelling evidence of DITP. Further work is needed to establish reliable, accessible diagnostic alternatives when advanced immunologic testing is unavailable. Overall, clinicians should not hesitate to prescribe clinically indicated medications, but they must maintain a high index of suspicion for DITP when the clinical context warrants it.
